# Achieving Body Weight Adjustments for Feeding Status and Pregnant or Non-Pregnant Condition in Beef Cows

**DOI:** 10.1371/journal.pone.0112111

**Published:** 2015-03-20

**Authors:** Mateus P. Gionbelli, Marcio S. Duarte, Sebastião C. Valadares Filho, Edenio Detmann, Mario L. Chizzotti, Felipe C. Rodrigues, Diego Zanetti, Tathyane R. S. Gionbelli, Marcelo G. Machado

**Affiliations:** 1 Federal University of Lavras, Department of Animal Science, Lavras, MG, Brazil; 2 Federal University of Viçosa, Department of Animal Science, Viçosa, MG, Brazil; INRA, FRANCE

## Abstract

**Background:**

Beef cows herd accounts for 70% of the total energy used in the beef production system. However, there are still limited studies regarding improvement of production efficiency in this category, mainly in developing countries and in tropical areas. One of the limiting factors is the difficulty to obtain reliable estimates of weight variation in mature cows. This occurs due to the interaction of weight of maternal tissues with specific physiological stages such as pregnancy. Moreover, variation in gastrointestinal contents due to feeding status in ruminant animals is a major source of error in body weight measurements.

**Objectives:**

Develop approaches to estimate the individual proportion of weight from maternal tissues and from gestation in pregnant cows, adjusting for feeding status and stage of gestation.

**Methods and Findings:**

Dataset of 49 multiparous non-lactating Nellore cows (32 pregnant and 17 non-pregnant) were used. To establish the relationships between the body weight, depending on the feeding status of pregnant and non-pregnant cows as a function of days of pregnancy, a set of general equations was tested, based on theoretical suppositions. We proposed the concept of pregnant compound (PREG), which represents the weight that is genuinely related to pregnancy. The PREG includes the gravid uterus minus the non-pregnant uterus plus the accretion in udder related to pregnancy. There was no accretion in udder weight up to 238 days of pregnancy. By subtracting the PREG from live weight of a pregnant cow, we obtained estimates of the weight of only maternal tissues in pregnant cows. Non-linear functions were adjusted to estimate the relationship between fasted, non-fasted and empty body weight, for pregnant and non-pregnant cows.

**Conclusions:**

Our results allow for estimating the actual live weight of pregnant cows and their body constituents, and subsequent comparison as a function of days of gestation and feeding status.

## Introduction

The breeding herd accounts for about 70% of the total energy used in beef cattle production [1]. However, the number of studies investigating the production efficiency of breeding cows is lower than the number of studies involving the same aspect in other categories of beef cattle production system such as growing and finishing animals [2]. One of the limiting factors for evaluating the production efficiency (e.g. feed efficiency) in mature cows is the difficulty to obtain reliable estimates of weight variation. This occurs due to the interaction of weight variation with specific physiological stages such as pregnancy and lactation.

In studies with pregnant cows both rate of body weight (**BW**) change and reproductive performance are commonly measured for assessment of the response of animals to experimental treatments. The deposition of body tissue reserves as well as fetal and uterine tissues contributes to the increase of BW of the cow leading to a complicated interpretation of the BW change. The comparison of the BW of a pregnant cow at the beginning and end of a study may not accurately represent the different physiological status because of the increased weight due to deposition of body tissue reserves or due to the growth of the components related to pregnancy, such as the gravid uterus and mammary gland. As such the standardization of BW of a cow in pregnant or non-pregnant condition is the first step to meet their nutrient requirements [3].

It is also noteworthy that cattle are known to vary weight in function of feeding status (fasting or fed) throughout the day. Variation in gastrointestinal contents in ruminant animals is a major source of error in body weight gain measurements [4]. Weighing forms typically known are fed BW, shrunk BW (**SBW**, weight after 14 to 16 hour fasting) and empty BW (**EBW**, body weight without gastrointestinal content). The EBW is the weight information that presents the greatest correlation to carcass and animal traits [5], but can be directly acquired only after slaughter. Thus, the first step to estimate the nutrient requirements of cattle is to know the correct relationship between the BW, SBW and EBW. The Beef Cattle NRC system [6] adopted suggestions for weight adjustments among BW, SBW and EBW for growing animals and adult cows but not for pregnant cows. The Dairy Cattle NRC [7] adopted fixed factors of adjustments among BW, SBW and EBW without considerations about age and physiological status of the animal, while the ARC [8] and AFRC system [9], however, does not suggest BW adjustments in function of feeding and physiological status of the animal. The INRA System [10] suggests an adjustment between BW and EBW based on the neutral detergent fiber content of the diet [11, 12]. Moreover, the correction factors for the weight of adult cows based on physiological condition in all feeding systems are minimal. Currently, there is no information with regard to the relationship between BW and SBW, and other relationship and adjustments for live weight of *Bos indicus* pregnant cows.

A few previous studies reported data about BW adjustments for pregnancy in cows [13, 14]. However, the available data did not consider a possible variation in the udder weight, which may increase in size as a function of pregnancy and consequently increases the weight of the cow. The use of equations and relationships to estimate the weight of the gravid and non-gravid uterus and the cow’s udder as a function of gestational age and other characteristics allows the estimation of the live weight of cows and their body constituents. Additionally, it also allows comparision of body weight at various physiological stages and chronological sequences. Thus, we aimed to develop approaches to estimate the actual weight of the adult cow regardless of feeding status (fasted or not) throughout pregnancy. We hypothesized the existence of an increase in the udder weight as a function of gestation and tested at which point of gestation this increase is significant. Our results provide an approach that allows to determine the individually contribution of maternal and gestational tissues or tissues that increased in size due to pregnancy to body weight of a live cow.

## Materials and Methods

### Ethics Statement

All animal procedures were approved by the Animal Care and Use Committee of the Department of Animal Science of the Universidade Federal de Viçosa—Brazil (047/2012).

### Animals

Forty-nine multiparous Nellore cows with average initial body weight of 451 ± 10 kg, age of 5.6 ± 0.5 years and body condition score of 4.4 ± 0.2 (1 to 9 scale) were used. Cattle used in the present study were from an experiment reported by Duarte et al. [15]. From the initial 49 cows, 32 were randomly separated and hand mated with Nellore bulls to form the pregnant group. Twelve cows were randomly separated and assigned to the non-pregnant group and the five remaining cows were designated as the baseline group.

### Diet and management

Cows were housed in pens (48 m^2^, 6 cows per pen) with a concrete floor, 15 m^2^ of covered area. Feed intake was measured individually using an electronic head gate system (Kloppen Soluções Tecnológicas, Pirassununga, SP, Brazil). Bred cows were at 47 ± 3 d of gestation at the beginning of the feeding trial.

All cows were fed the same diet twice daily (0700 and 1500 h) and divided into two groups according feeding level (**FL**), either **HIGH** (*ad libitum*) or **LOW** (restricted feeding 1.2-times maintenance according to the Beef Cattle NRC [6]). Sixteen pregnant and 5 non-pregnant cows were fed HIGH level and 16 pregnant and 7 non-pregnant cows were fed the LOW-diet. The restricted feeding was estimated so that 1.2-times maintenance sustained pregnancy and the HIGH-fed allowed maternal tissue deposition. A high digestible diet based on corn silage, ground corn, soybean meal, urea and mineral mixture (Table 1) was used to allow the cows to have sufficiently different weight gain and body condition score (**BCS**) among the nutritional. Intake was measured by the difference between the offered and the orts (samples of the offered feeds and orts were collected every week for dry matter analysis by AOAC Method 934.01 [16]). Cattle had *ad libitum* access to water.

**Table 1:** Ingredients and chemical composition of the diet.

Item	Silage	Concentrate	Diet
Ingredient, % of DM			
Corn Silage	100.0	-	84.3
Ground corn	-	54.6	8.5
Soybean meal	-	33.0	5.1
Urea	-	7.3	1.2
Sodium chloride	-	2.1	0.37
Ammonium sulfate	-	1.5	0.25
Dicalcium phosphate	-	1.3	0.23
Microminerals mixture^1^	-	0.17	0.028
Analyzed composition^2^, %			
DM	28.0	89.2	37.6
OM	94.7	92.8	94.4
CP	7.8	44.1	13.5
EE	2.9	2.5	2.8
NDF_ap_	45.8	8.2	39.9
iNDF	20.8	0.65	17.6
NDIN	38.2	7.0	11.4
NFC	38.2	53.0	40.6
TDN	-	-	66.6
GE (Mcal/kg)	3.82	3.49	3.77

^1^Zinc sulfate (56.3%), manganese sulfate (26.2%), copper sulfate (16.8%), potassium iodate (0.37%), cobalt sulfate (0.23%) and sodium selenite (0.10%).

^2^NDF_ap_ = neutral detergent fiber corrected to ash and protein, iNDF = indigestible neutral detergent fiber and NFC = non fibrous carbohydrates.

Every 28 days cows were weighed in the morning (0700 h, before feeding) to obtain the BW, and reweighed at the same time the following day after 16 h of solids fasting to obtain the SBW values. Thus, the body gain (**BG**, kg/d) and the shrunk body gain (**SBG**, kg/d) were calculated.

The 32 pregnant cows were randomly assigned into 4 groups of 8 cows each (4 cows per each feeding level) and slaughtered at 136 ± 1, 189 ± 1, 239 ± 1 and 269 ± 1 days of pregnancy (**DOP**). Non-pregnant cows were slaughtered at different times during the experiment (85 to 216 days of feeding control) to keep them in experiment for a similar period of time as the pregnant cows. For regression analysis as a function of DOP, the non-pregnant cows were considered as DOP = 0. Cows from the baseline group were harvested on day 0 of the experiment after the end of the adaptation period and were used to estimate the initial BW, SBW and EBW of the cows in the experiment.

### Animal harvest and weighing of uterus and udder

Pre-slaughter animal care and handling procedures followed the Sanitary and Industrial Inspection Regulation for Animal Origin Products [17]. Animals were submitted to a 16 h fasting prior to slaughter with free access to water. Slaughter was performed by using captive bolt stunning and exsanguination. The euthanasia of fetuses followed the American Veterinary Medical Association Guidelines [18].

Udders were removed along with the portion of hide that covered the udder and then weighed immediately after slaughter. The gravid and non-gravid uteri were sectioned at the cervix and weighed. Digestive tract were separated into parts and then washed to remove gastrointestinal contents. The washed digestive tract parts and the whole body components were weighed and to obtain the value of empty body weight (**EBW**_**p**_, including udder and gravid uterus of pregnant cows) or the non-pregnant empty body weight (**EBW**_**np**_, for non-pregnant cows).

### Body condition score

Body condition scores were assessed on a scale ranging from 1 = severely emaciated to 9 = very obese [19, 20] with 0.5 partial scoring and were determined by observation and palpation. The trial for estimating the BCS of each cow was performed on the day of slaughter by two trained evaluators in a double-blind scheme. Each evaluator did not know the result of the evaluation of the other, and the average score was calculated. A new evaluation was performed if the scores of the two evaluators were further than 1.5 points apart.

### Adjustment of cow BW measurements

To establish the relationships between the BW, SBW and EBW of pregnant and non-pregnant cows as a function of time of gestation, a set of general equations was tested, based on theoretical suppositions presented and explained in Table 2. In general, the pregnancy (referred mathematically as pregnancy component, **PREG**) was mathematically considered as an extra component of the cow (cow BW + PREG). However, it was assumed the existence of an interaction between the development of the specific tissues for the pregnancy (gravid uterus and udder) and the maternal tissues (lean, fat, viscera, etc). When appropriate, this interaction was considered and tested.

**Table 2:** Set of general theoretical assumptions used to establish cows BW adjustments.

Code	Model	What it means
A1	SBW_np_ = ƒ(BW_np_)	SBW of a non-pregnant cow (**SBW**_**np**_) is function of the BW of a non-pregnant cow (**BW**_**np**_)
A2	SBW_p_ = ƒ(BW_p_, DOP)	SBW of a pregnant cow (**SBW**_**p**_) is function of BW of a pregnant cow (**BW**_**p**_) and days of pregnancy (**DOP**)
A3	SBW_p_ = SBW_np_ + PREG	SBW_p_ also can be expressed as the SBW of the cow if in a non-pregnant condition plus the increase of weight occurred due to the pregnancy, called pregnancy compound (**PREG**). For this we need to consider non interaction between the ratio BW/SBW and DOP
A4	SBW_np_ = SBW_p_—PREG	SBW_np_ if the cow is pregnant can be estimated as the SBW_p_ minus PREG. Is the inverse of the equation A3
B1	EBW_np_ = ƒ(SBW_np_)	EBW of a non-pregnant cow (**EBW**_**np**_) is function of SBW of a non-pregnant cow (**SBW**_**np**_)
B2	EBW_p_ = ƒ(SBW_p_, DOP)	EBW of a pregnant cow (**EBW**_**p**_) is function of SBW_p_ and DOP
B3	EBW_p_ = EBW_np_ + PREG	EBW_p_ also can be expressed as the EBW of the cow if in a non-pregnant condition plus the pregnancy compound. For this we need to consider that when discounted the PREG, the relation between SBW and EBW is equal for pregnant and non-pregnant cows
B4	EBW_np_ = EBW_p_—PREG	EBW_np_ if the cow is pregnant can be estimated as the EBW_p_ minus PREG. Is the inverse of the equation B3
C	PREG = GU_dp_ + UD_dp_	PREG means the all tissues increase due to the pregnancy and is equal to the GU accretion during the pregnancy (**GU**_**dp**_) plus udder accretion during the pregnancy (**UD**_**dp**_)
D	GU_dp_ = GU—UT_np_	GU_dp_ is equal to GU minus the weight of the uterus of the cow in non-pregnant condition (**UT**_**np**_)
E	GU = fetus + amniotic fluid + placenta + uterus + ovaries	Gravid uterus (**GU**) is equal to the sum of its compounds
F	GU = ƒ(SBW, FL, DOP, CBW)	GU is function of SBW, feeding level (**FL**), DOP and calf birth weight (**CBW**)
G	UT_np_ = ƒ(SBW)	UT_np_ is function of SBW
H	UD_dp_ = UD_p_—UD_np_	UD_dp_ is equal to the weight of udder of a pregnant cow (**UD**_**p**_) minus the udder weight of the cow in a non-pregnant condition (**UD**_**np**_)
I	UD_p_ = ƒ(SBW, FL, DOP)	UD_p_ is function of SBW, FL and DOP
J	UD_np_ = ƒ(SBW, FL)	UD_np_ is function of SBW and FL

Multiple linear regressions (for Eq. A2, B2, F, I and J of Table 2), simple linear regressions (for Eq. A1, B1 and G of Table 2), or non-linear regressions were used to test the relationships between BW, SBW, and EBW. The intercept was used when biologically appropriate and statistically significant.

The basic mathematical model used to estimate the dynamics of gravid uterus (**GU**) as a function of time of pregnancy was of the logistic form, similar previously suggested by Koong et al. [21]:

$$GU=GU_{0}\times e^{(\beta_{1}+\beta_{2}\times DOP+\beta_{3}\times BCS)\times DOP}$$ <1>

### Equation {1}

where DOP = days of pregnancy, BCS = body condition score, GU_0_ = weight of gravid uterus (kg) on day 0 of gestation and GU = the weight of gravid uterus (kg) on day DOP of pregnancy.

The mathematical model used to estimate the dynamics of udder as a function of time of gestation was based on previously reports from Fadel [22], as a segmented non-linear model with a period of staticity followed by an exponential model, as presented bellow:

If DOP < *β*_*0*_ then

$$UD=SBW_{np}\times \beta_{1}\times BCS^{\beta_{2}}$$ <2>

If DOP > *β*_*0*_ then

$$UD=SBW_{np}\times \beta_{1}\times BCS^{\beta_{2}}\times e^{\left[\beta_{3}\times \left(DOP-\beta_{0}\right)\right]}$$ <3>

where: DOP = days of pregnancy, UD = udder (kg), SBW_np_ = shrunk body weight of a cow in non-pregnant condition (kg) and BCS = body condition score. The parameter *β*_*0*_ represents the moment in which the weight of the udder starts to increase as a function of days of pregnancy. The model <2> is used to estimate the weight of the udder of non-pregnant cows and the use of <2> or <3> model is used to pregnant cows depending on the values of *B*_*0*_ and DOP.

### Statistical Analysis

Feed intake, initial and final SBW and BCS of the cows were analyzed through a model including the fixed effects of feeding level (**FL**, HIGH or LOW), DOP (non-pregnant, 136, 189, 239 and 269 days of pregnancy) and their interaction as it follows:

$$Y_{ijk}=\mu +FL_{i}+DOP_{j}+{\left(FL\times DOP\right)}_{ij}+e_{ijk}$$ <4>

where FL_*i*_ is the *i*th level of the fixed effect of FL, DOP_*j*_ is the *j*th level of the fixed effect of DOP, and *e*_*ijk*_ is the random error associated with *Y*_*ijk*_.

The REG and GLM procedures of SAS version 9.2 (SAS Inst. Inc., Cary, NC) were used to estimate the regression parameters of linear functions. Parameters for nonlinear functions were estimated using the Gauss-Newton method in the NLIN procedure of SAS version 9.2.

The value of 0.05 was adopted as critical level of probability for occurrence of Type I error. Trends were identified when 0.05<*P*≤0.10. Data are presented as least square means ± pooled standard errors for means and estimated parameter ± standard error for parameters of linear and non-linear models.

### Model comparisons

When relevant, two predictive models with different structure using the same database were compared. The comparisons were based on the significance of the regression between the observed and predicted values [23], residual analysis, accuracy (**Cb**) [24], the mean square error of prediction (**MSEP**) [25] and the partition of sources of variation of MSEP [26]. These statistics were calculated using the Model Evaluation System (MES, v.3.0.1, http://nutritionmodels.tamu.edu/mes.htm) [27]. In addition, the Akaike Information Criteria (**AIC**) [28] was calculated according to Motulsky and Christopoulos [29].

### Cross-validation of the models

All fitted statistical models adjusted were evaluated by the cross-validation [30] as a validation procedure. The SURVEYSELECT procedure of SAS version 9.2 was used to create the cross validation samples. For each model 20 replicates (sub samples of the original dataset) were randomly created using 70% of the data each. The OUTALL option was used to tell the procedure to output all of the records from the input dataset, but to mark the selected sample records. For this, a new variable (SELECTED) was created (SELECTED was 1 for the chosen records and 0 for the rest). The model was then fitted using only the selected data by replicate and predicted values for response variables were created. Deviations (***d***) between predicted and observed values were calculated for each replicate and used to estimate the Root Mean Square Error (**RMSE**, standard error of *d*) and the mean absolute error (**MAE**, absolute mean of *d*) for each model. Correlations (**R**) between predicted and observed values were estimated and the R^2^ in the test was calculated as the square of R values.

All predicted statistic parameters are provided with at least three significant digits. Models comparisons and discussions are made separately for each sub section while models validation (by cross-validation) is presented and discussed in a specific sub section.

## Results and Discussion

### Intake, SBW, SBG and BCS at slaughter

At the beginning of the experiment cows had similar SBW between the feeding groups (*P* = 0.98) and gestational period groups (*P* = 0.83), averaging 436 ± 15 kg (Table 3). During the trial, HIGH-fed cows consumed approximately 1.65 times (*P*<0.01) the amount of food that was consumed by LOW-fed cows (7.62 *vs* 4.60 kg/day for HIGH and LOW-fed cows, respectively). Consequently, the HIGH-fed cows had greater SBW, SBW of maternal tissues only (**SBW**_**np**_), SBG, SBG of maternal tissues only (**SBG**_**np**_) and BCS (*P*<0.01, Table 3). Due to feeding levels applied the SBW and BCS of the cows ranged from 366 kg and 3.0 to 701 kg and 8.0, respectively. These variations were crucial to improve the accuracy of adjustment of linear and non-linear models of pregnancy compounds as a function of cow’s weight and BCS.

**Table 3:** Intake, body change and body condition score of pregnant and non-pregnant Nellore cows as a function of days of pregnancy and feeding level.

Item^1^	Day of pregnancy					Feeding level^2^		P-value^3^		
	Empty	136	189	239	269	High	Low	FL	DOP	FL×DOP
	(n = 12)	(n = 8)	(n = 8)	(n = 8)	(n = 8)	(n = 21)	(n = 23)			
DMI, kg/day	6.00 ± 0.35	6.28 ± 0.41	6.20 ± 0.41	6.13 ± 0.41	5.94 ± 0.41	4.60 ± 0.16	7.62 ± 0.16	<0.01	0.77	0.28
iSBW, kg	435 ± 20	454 ± 24	422 ± 24	448 ± 24	423 ± 24	436 ± 15	437 ± 14	0.98	0.83	0.81
fSBW, kg	497 ± 20	508 ± 26	494 ± 26	555 ± 26	551 ± 26	563 ± 16	480 ± 16	<0.01	0.25	0.39
High	535^de^ ± 11	515^cd^ ± 13	554^e^ ± 13	591^f^ ± 13	623^g^ ± 13	-	-	-	-	-
Low	467^a^ ± 10	468^a^ ^b^ ± 13	464^a^ ± 13	498^b^ ^c^ ± 13	506^c^ ± 13	-	-	-	-	-
rfSBW, kg	501 ± 7	484 ± 9	495 ± 9	509 ± 9	518 ± 9	543 ± 5	460 ± 5	<0.01	<0.01	0.07
High	535^bc^ ± 11	508^b^ ± 12	540^c^ ± 12	558^cd^ ± 12	572^d^ ± 12	-	-	-	-	-
Low	467^a^ ± 10	459^a^ ± 12	450^a^ ± 12	461^a^ ± 12	464^a^ ± 12	-	-	-	-	-
SBG, kg/day	0.54 ± 0.05	0.62 ± 0.06	0.48 ± 0.06	0.59 ± 0.06	0.58 ± 0.06	0.86 ± 0.04	0.26 ± 0.04	<0.01	0.60	0.98
rSBG, kg/day	0.54^c^ ± 0.05	0.53^b^ ^c^ ± 0.06	0.38^a^ ± 0.06	0.40^a^ ^b^ ± 0.06	0.37^a^ ± 0.06	0.73 ± 0.04	0.15 ± 0.04	<0.01	0.07	0.95
iBCS	4.4 ± 0.3	4.6 ± 0.4	4.4 ± 0.4	4.6 ± 0.4	4.3 ± 0.4	4.7 ± 0.2	4.2 ± 0.2	0.09	0.94	0.31
fBCS	5.5^a^ ^b^ ± 0.2	5.3^a^ ± 0.2	5.8^b^ ^c^ ± 0.2	6.2^cd^ ± 0.2	6.3^d^ ± 0.2	6.5 ± 0.2	5.1 ± 0.2	<0.01	<0.01	0.55
BCSG	0.88 ± 0.11	0.88 ± 0.13	0.88 ± 0.13	0.95 ± 0.13	0.81 ± 0.13	1.40 ± 0.08	0.36 ± 0.08	<0.01	0.96	0.98

^a-b^Within a variable, means differ (P<0.05).

^1^DMI = dry matter intake, iSBW = initial shrunk BW, fSBW = final shrunk BW, rfSBW = real final shrunk body weight (discounting the weight of tissues related to the gestation), SBG = shrunk body gain, rSBG = real shrunk body gain (discounting the weight of tissues related to the gestation), iBCS = initial body condition score (1 to 9 scale), fBCS = final body condition score, BCSG = body condition score gain (points of BCS per each 100 days of experiment).

^2^High = HIGH-fed cows and Low = LOW-fed cows.

^3^Probability values for effects of feeding level (FL), day of pregnancy (DOP), and their interaction (FL × DOP).

A trend for significant interaction between feeding level and DOP was observed (*P* = 0.07) on the SBW_np_ at slaughter. Despite of the maternal tissues accretion (average 0.15 kg/day) observed in LOW-fed cows, no significant variation was observed for SBW_np_ (*P*>0.05) between the non-pregnant and pregnant cows and between pregnant cows at different stages of pregnancy (Table 3), while HIGH-fed cows increased SBW_np_ during the pregnancy (*P*<0.05).

### Estimation of SBW from BW

The BW and DOP were significant (*P*<0.05) to estimate the SBW of pregnant and non-pregnant cows when a multiple linear regression was used (Table 2, Eq. A1 and A2). However, less than 0.5% of the variation in the SBW of the pregnant cows may be explained by DOP. The AIC was greater when the BW and DOP were used in the model (AIC = 263.4) than when only BW was used (AIC = 258.4). The theoretical models presented in the Eq. A3 and A4 (Table 2) cannot be practically developed because the estimative of PREG is dependent on the SBW value (Table 2, Eq. C, D and F), making it impractical to consider a possible interaction between the SBW/BW ratio and the time of pregnancy. Thus, we decided to use only BW to estimate SBW of pregnant and non-pregnant cows, assuming that the effect of DOP is negligible.

The equations generated to predict the SBW only as a function of the BW (Equation <5> and <6>) had high Cb values (0.999 for Equation <5> and 1.000 for Equation <6>) when the estimated and observed data were compared. However, the hypothesis that β_0_ = 0 and β_1_ = 1 was rejected (P = 0. 003) for Equation <5> when the parameters were analyzed. For the model presented in Equation <6>, this hypothesis was accepted (P = 0.889). Equation <6> also has a lower MSEP (23.6) than the linear model (28.9). Moreover, the decomposition of the MSEP shows that only 0.06 (0.3%) of the MSEP value originated from the systematic errors in the power model (Equation <6>), whereas in the linear model this value is 4.62 (15.9% of the MSEP). The AIC also shows a better value for the power model in comparison with the linear model (250.5 and 258.4, respectively). This difference indicates that, according to the AIC theory (based on the AIC evidence ratio) [29], there is a 98.2% of probability that the model given by Equation <6> is better than that from Equation <5>. This means that the model Equation <6> of is 53-times more likely to be correct than the Equation <5> model.

These results suggest the utilization of a nonlinear model to estimate the SBW value for pregnant and non-pregnant cows. The value of the BW exponent greater than 1 (1.0303) shows an increase in the value of the relationship between SBW and BW as body weight increases. Biologically, it suggests that the decrease of weight caused by fasting is lower in proportion to the whole body weight as BW increases. However, when the variations in the BW of cows herd occur due to higher variations in age or number of lactations the use of a model with a variable effect of BW on SBW may be not adequate. In this case, the use of a fixed relation (Equation <5>) is suggested. Based on this, although the fit of the model given by Equation <6> is better than from Equation <5>, both models were kept as the result of this study and the choice of the use of each will depend on the characteristics of the cow herd.

SBW=0.9763(±0.0013)×BW <5>

$$SBW=0.8084\left(\pm 0.0350\right)\times BW^{1.0303\left(\pm 0.0069\right)}$$ <6>

where SBW = shrunk body weight (kg) and BW = body weight (kg).

The relationship between SBW and BW calculated using the Equation <6> for a 300-kg BW cow is 0.961, near to the value suggested by the Beef Cattle NRC [6], which is 0.96. However, for a 700kg BW cow this relationship is equal to 0.986.

### Gravid uterus weight

Theoretically, the weight of the GU would be a function of weight of the cow (SBW), the feeding level, and the time of pregnancy. The inclusion of SBW in the model is unworkable, because the weight of the GU is being estimated to estimating the cow SBW. Likewise, although differences have been observed in the weight of the GU at 270 days of gestation as a function of feeding level with the same cows used in this study [2], the inclusion of feeding level in the model is impractical because the feeding level is a variable concept. If feeding level refers to daily intake of DM (kg/day), there may have differences in energy concentration among diets. If feeding level is referred as the proportion of maintenance energy level, the concept cannot be practically applied because the energy requirements of Zebu beef cows are still unclear. Thus, we decided to use of BCS as it is easy to measure and indirectly includes information about cow weight and feeding level. When the DOP and BCS where included in the model it was better fitted to the data than when DOP and feeding level were included (AIC = 52.0 and 54.7; MSEP = 23.6 and 28.8, for inclusion of DOP and BCS or DOP and feeding level, respectively). In case of no availability of the BCS, a model to predict the GU as function only of DOP was also generated (Equation <8>).

$$GU=0.2243\left(\pm 0.3099\right)\times BCS^{0.3225\left(\pm 0.1331\right)}\times e^{\left(\left(0.02544\left(\pm 0.0125\right)-0.0000286\left(\pm 0.000028\right)\times Dop\right)\times Dop\right)}$$ <7>

$$GU=0.2106\left(\pm 0.3238\right)\times e^{\left(\left(0.03119\left(\pm 0.0137\right)-0.00004117\left(\pm 0.00003\right)\times Dop\right)\times Dop\right)}$$ <8>

where GU = gravid uterus (kg), DOP = days of pregnancy and BCS = body condition score.

The estimation of GU weight can be scaled from the estimated calf birth weight (**CBW**). The estimated CBW has been used to estimate the nutrient requirements for pregnancy in *Bos taurus* cattle [6] and to adjust the BW of the dam [31] in dairy cattle. Based on this, we suggest the use of GU weight as a proportion of estimated CBW. The use of an estimated GU weight scaled for CBW is important when a set of equations from other breed or from crossbred Zebu cattle in the model presented in this study, once the CBW is known to be different between *B*. *indicus* and *B*. *taurus* cattle [32]. The estimated fetus weight at 290 days of gestation (average length of gestation in *B*. *indicus* cattle, [33]) in this study was 28 kg [2], while in the study of Ferrell et al. [34], which was performed using *B*. *taurus* cows, the average estimated CBW was 38.5 kg.

Thus, the models presented as <7> and <8> were scaled for a CBW of 28 kg and the models <9> and <10> were generated:

$$GU=0.008010\left(\pm 0.0111\right)\times CBW\times BCS^{0.3225\left(\pm 0.1331\right)}\times e^{\left(\left(0.02544\left(\pm 0.0125\right)-0.0000286\left(\pm 0.000028\right)\times DOP\right)\times DOP\right)}$$ <9>

$$GU=0.007521\left(\pm 0.0116\right)\times CBW\times e^{\left(\left(0.03119\left(\pm 0.0137\right)-0.00004117\left(\pm 0.00003\right)\times DOP\right)\times DOP\right)}$$ <10>

where GU = gravid uterus (kg), CBW = calf birth weight (kg), DOP = days of pregnancy and BCS = body condition score.

The Table 4 shows examples of weights of GU estimated by Equation <9> and <10> for cows with different BCS in different times of pregnancy.

**Table 4:** Examples of the estimation of gravid uterus weight as a function of days of pregnancy (Equation <9>) or days of pregnancy and body condition score (<Equation 10>) for an estimated calf birth weight of 28 kg in Zebu cows.

Equation	Body condition score		
	3	5	7
*135 d in pregnancy*			
<9>	6.71	6.71	6.71
<10>	5.88	6.94	7.73
*270 d in pregnancy*			
<9>	47.6	47.6	47.6
<10>	38.2	45.0	50.2

### Non-pregnant uterus and ovaries weight

A linear model without intercept showed the best fit (based on AIC value) to describe the weight of the uterus plus ovaries of non-pregnant cows (**UT**_**np**_, <Equation 11>). The same model can be used to estimate the UT_np_ of pregnant cows. However, an adjustment to the value of SBW (<Equation 12>) is required, which is an adaptation for a provisional value of SBW_np_ of pregnant cows as there is a circular reference at this point due to the fact that the estimate of UT_np_ is part of the estimates made to obtain the real value of SBW_np_.

$$UT_{np}=0.0012\left(\pm 0.00005\right)\times SBW_{np}$$ <11>

$$\begin{array}{l} if\;DOP\leq 240\;then\;UT_{np}=0.0012\left(\pm 0.00005\right)\times \left(SBW_{p}-GU+0.6\right)\\ if\;DOP>240\;then\;UT_{np}=0.0012\left(\pm 0.00005\right)\times \left(SBW_{p}-GU+0.6-2\right) \end{array}$$ <12>

where UT_np_ = uterus plus ovaries of a non-pregnant cow (kg), SBW_np_ = non-pregnant shrunk body weight, SBW_p_ = pregnant shrunk body weight, 0.6 = the value of a UT_np_ of a 500-kg cow and 2 = the average value for udder accretion due to pregnancy when pregnancy is longer than 240 days. The coefficients of 0.6 and 2 were used because it is not possible at this point to estimate the exact value of SBW_np_ for a pregnant cow. Thus, they were used as estimates to produce the minimum error as possible.

### Accretion of weight as gravid uterus during pregnancy

From the values of GU and UT_np_ the accretion of weight as gravid uterus during pregnancy (**GU**_**dp**_) can be estimated according to Eq. D of Table 2. As an example, for a 500-kg BW pregnant cow, with BCS of 5, 200 days of pregnancy and with an estimated CBW of 28 kg, the calculation of GU_dp_ is:

$$\begin{array}{l} GU=0.008010\times 28\times 5^{0.3225}\times e^{\left(\left(0.02544-0.0000286\times 200\right)\times 200\right)}=19.44kg\\ GU_{dp}=GU-UT_{np}\\ GU_{dp}=19.44-0.0012\times \left(500-19.44+0.6\right)\\ GU_{dp}=18.86kg \end{array}$$

### Udder modeling

**Fig 1:**
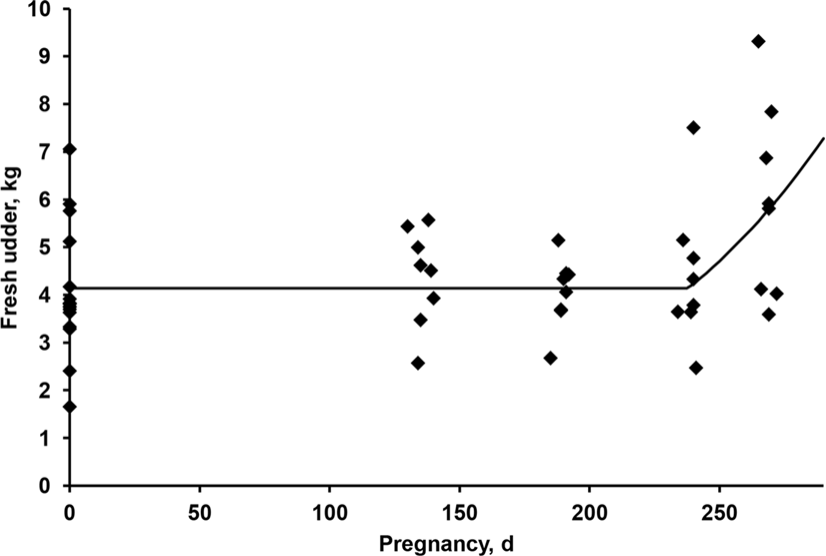
Relationship between days of pregnancy and weight of fresh udder in Nellore cows. The continuous line represents the estimation of the weight of fresh udder for a cow with the average shrunk body weight and body condition score (494 kg and 5.6, respectively) of the cows used in this study.

During pregnancy, mainly near parturition, changes occur in the bovine mammary gland, through the formation of milk secretory tissue. Although this phenomenon commonly occurs in heifers, this was also observed in multiparous cows used in the current study. The udder weight data showed an increase in the weight from 240 days of pregnancy (Fig. 1). The non-linear logistic model (Equation <1>) did not fit the data of udder weights well as a function of DOP. Thus, a non-linear segmented model (Eq. [22]), as presented by Fadel [23], was used to describe the dynamics of the udder in function of gestation. The model that showed better fit was with the inclusion of SBW, BCS and DOP for pregnant cows and SBW and BCS for non-pregnant cows (<Equation 13>). The intercept of the model was 238, indicating no significant increase in the weight of the cow’s udder at this period of pregnancy. From this period, there is an exponential increase in udder weight due to the formation of parenchyma. Similarly to the weight of the gravid uterus, the feeding level was not included, but is indirectly represented by the BCS. The SBW_np_ was included in this model because it represents much of the variation in the weight of the udder. As previously observed there is also a case of a circular reference, and to estimate the SBW_np_ of pregnant cows it is suggested to use SBW_np_ = SBW_p_—GU + 0.6 (and—2 if DOP is greater than 240 days).

$$\begin{array}{l} if\;DOP\leq 238\;then\;UD_{np}=SBW_{np}\times 0.00589\left(\pm 0.00192\right)\times BCS^{0.2043\left(\pm 0.1809\right)}\\ if\;DOP>238\;then\;UD_{p}=UD_{np}\times e^{\left(\left(DOP-238\right)\times 0.0109\left(\pm 0.0019\right)\right)} \end{array}$$ <13>

where **UD**_**np**_ = udder of a non-pregnant cow (kg), SBW_np_ = shrunk body weight of a non-pregnant cow (kg), BCS = body condition score, DOP = days of pregnancy, **UD**_**p**_ = udder of a pregnant cow (kg).

The high variability of udder weight among the cows complicates the modeling of factors that affect the weight of this component in beef cows. The model presented in <Equation 13> showed the best fit among other tested and so was used. When the observed values were regressed as a function of predicted values, a Cb = 0.9926 and MSEP = 0.642 were obtained, being 92.8% of MSEP variations from random sources. The hypothesis that β_0_ = 0 and β_1_ = 1 was tested according to Mayer et al. [6] and was accepted (P = 0.209), indicating that the model is appropriate.

Based on the model presented in <Equation 13> the increase in udder in function of pregnancy (**UD**_**dp**_, Eq. H in Table 2) can be defined as follows:

$$\begin{array}{l} if\;DOP\leq 238thanUD_{dp}=0\\ if\;DOP>238thanUD_{dp}=UD_{np}\times e^{\left(\left(DOP-238\right)\times 0.0109\left(\pm 0.0019\right)\right)}-UD_{np} \end{array}$$ <14>

where DOP = days of pregnancy and UD_dp_ = udder accretion due to pregnancy. In practice, it is suggested the adoption of 240 days of pregnancy as the time in which the udder starts to increase due to pregnancy.

### Pregnancy compound

Based on the Eq. C of the Table 2, the pregnant compound (**PREG**) can be defined as Equation <9>–<Equation 12> + <Equation 14>, as it follows:

$$\begin{array}{l} PREG=GU_{dp}+UD_{dp}\\ PREG=GU=UT_{np}+UD_{dp} \end{array}$$

If the pregnancy is less than 240 days the value of UD_dp_ must be excluded.

As an example, a pregnant cow with 550 kg of SBW_p_, BCS of 6, estimated CBW of 28 kg at 270 d of pregnancy, the BW that is attributable to the pregnancy can be calculated as it follows:

$$\begin{array}{l} PREG=GU_{dp}+UP_{dp}\\ GU_{dp}=GU-UT_{np}\\ GU=0.008010\times CBW\times BCS^{0.3225}\times e^{\left(\left(0.02544-0.0000286\times DOP\right)\times DOP\right)}\\ GU=0.008010\times 28\times 6^{0.3225}\times e^{\left(\left(0.02544-0.0000286\times 270\right)\times 270\right)}\\ GU=47.8kg\\ GU_{dp}=GU-0.0012\times \left(SBW_{p}-GU+0.6-2\right)\\ GU_{dp}=47.8-0.0012\times \left(550-47.8+0.6-2\right)\\ GU_{dp}=47.2kg\\ UD_{dp}=UD_{np}\times e^{(\left(DOP-238\right)\times 0.0190}-UD_{np}\\ UD_{np}=SBW_{np}\times 0.00589\times BCS^{0.2043}\\ UD_{np}=\left(550-47.8+0.6-2\right)\times 0.00589\times BCS^{0.2043}\\ UD_{np}=4.25kg\\ UD_{dp}=4.25\times e^{(\left(270-238\right)\times 0.0109}-4.25\\ UD_{dp}=1.76kg\\ PREG=47.2+1.76kg\\ PREG=49.0kg \end{array}$$

### Estimation of non-pregnant SBW of a pregnant cow

The knowledge of the values of SBW_p_ and PREG allows the estimation of the SBW_np_ values according to Eq. A4 (Table 2). For the cow in the previous example, the SBW_np_ value is: 550–49 = 501 kg. Therefore, in this case tissues related to pregnancy represented 8.9% of cow’s weight.

### Estimation of EBW from SBW

The fit of models to predict EBW from SBW was performed in a dataset that grouped the data from non-pregnant cows with SBW and EBW data of pregnant cows, from which was deducted the value of PREG, estimated as described above.

**Fig 2:**
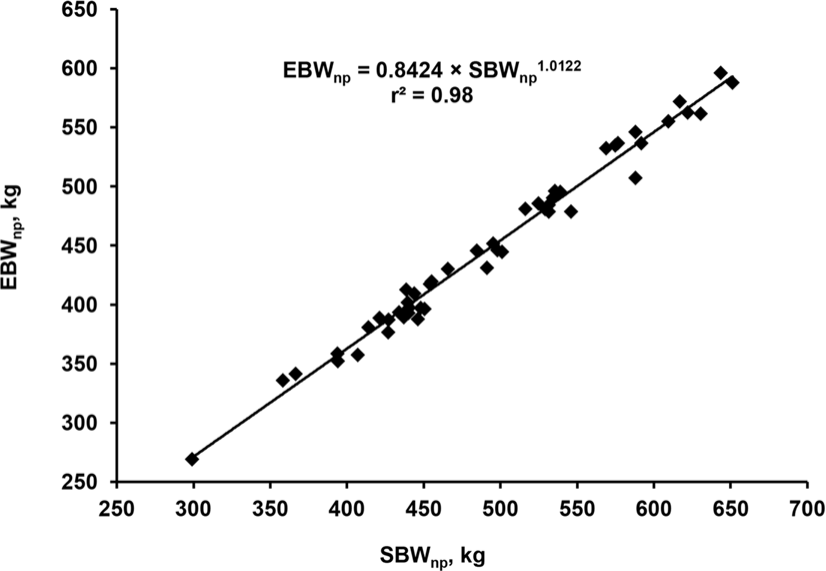
Relationship among non-pregnant shrunk body weight and non-pregnant empty body weight in Nellore cows. The continuous line represents the estimation of non-pregnant empty body weight from non-pregnant shrunk body weight using <Equation 16>.

The two equations generated to predict the EBW_np_ as a function of the SBW_np_ (<Equation 15> and <16>) have a Cb value of 0.999 for <Equation 15> and 1.000 for <Equation 16> when the estimated and observed data were compared. The hypothesis that β_0_ = 0 and β_1_ = 1 was accepted for both equations (P = 0.848 for <Equation 15> and P = 0.999 for <Equation 16>). <Equation 16> has a little lower MSEP (91.9) than the linear model (92.8). The decomposition of the MSEP shows that 99.2 and 100% of the MSEP value originates from random errors in <Equation 15> and <Equation 16>, respectively. The AIC was 98.4 for the <Equation 15> model and 100.2 for the <Equation 16> model. These results show that both models can be safely used to predict EBW_np_. However, we suggest the use of the power model (<Equation 16>) to have greater biological sense. A value of the SBW_np_ exponent greater than 1 (1.0122) means that there is an increase in the value of the relationship between EBW_np_ and SBW_np_ as body weight increases. This is biologically plausible and suggests that larger mature cows within the same breed have a lower proportion of weight on the content of the gastrointestinal tract by having logically lower proportion of the gastrointestinal tract.

$$EBW_{np}=0.9092\left(\pm 0.0028\right)\times SBW_{np}$$ <15>

$$EBW_{np}=0.8424\left(\pm 0.1025\right)\times SBW_{np}{}^{1.0122\left(\pm 0.0195\right)}$$ <16>

where EBW_np_ = empty body weight of a non-pregnant cow (kg) and SBW_np_ = shrunk body weight of a non-pregnant cow (kg). A graphic representation of the relation among EBW_np_ and SBW_np_ is shown in Fig. 2.

It should be noted observed that the relationship between EBW_np_ and SBW_np_ has a low variability between animals from different sizes or different feeding systems. The Beef Cattle NRC [35] and BR-CORTE [6] suggest the use of relations between EBW and SBW for growing animals of 0.891 and 0.895, respectively, similarly to those observed in the current study for adult cows. The relation between EBW_np_ and SBW_np_ calculated using <Equation 16> for a 300-kg cow is 0.9031 and for a 700-kg cow is 0.9125.

From the relationship between EBW_np_ and SBW_np_, the relationship between EBW_p_ and SBW_p_ can also be estimated. For this, it is necessary to deduct the value of PREG from these values. Based on Eq. B3 and Eq. B4 of Table 2, and <Equation 16>, the relationship between EBW_p_ and SBW_p_ can be calculated as it follows:

$$\begin{array}{l} EBW_{p}=EBW_{np}+PREG\\ EBW_{np}=0.8424\times SBW_{np}{}^{1.0122}\\ SBW_{np}=SBW_{p}-PREG,thus\\ EBW_{p}=\left(0.8424\times {\left(SBW_{p}-PREG\right)}^{1.0122}\right)+PREG \end{array}$$

The relationship consists of deducting the PREG component from the analysis, considering it as a separate portion of the weight of the cow. Based on the equations above the relationship between EBW_p_ and EBW_np_, and EBW_p_ and SBW_np_ can also be established.

### Model validation

The results of cross-validation procedure applied to the generated models are summarized in Table 5. The values of RMSE of models generated to predict SBW and EBW were lower and ranged from 1.02 to 1.09% of the mean for SBW predictors and from 2.12 to 2.13% of the mean for EBW predictors. The average correlation (R) and R^2^ between predicted and observed values of all 20 replicates of each model were higher (R≥0.992 and R^2^≥0.984) for SBW and EBW predictor models. This suggests that the usage of these models (Equation <5>, <6>, <15> and <16>) can be safely applied to the population were the cows used in this study come from. The variation in X-axis of SBW (299 to 701 kg) and EBW (269 to 596 kg) values is also a good indicator of accuracy in the fit of these models. The cross-validation results also corroborate with the model comparison results (MSEP, AIC and Cb), as previously presented, in which the non-linear models are usually a bit better than linear models to estimate the relationship between live weights at different feeding status of mature cows.

**Table 5:** Summary of cross-validation statistics from the predictive models generated.

Evaluated functions^1^			Statistics^2^								
Code	Predicted variable	Predictive variables	n	Mean ± SD	Minimum	Maximum	RMSE	RMSE (% of Mean)	MAE	R ± SD	R^2^
<5>—linear	SBW, kg	BW	173	505 ± 83	299	701	5.48	1.09	4.26	0.997 ± 0.0002	0.996
<6>—non-linear	SBW, kg	BW	173	505 ± 83	299	701	5.18	1.02	4.02	0.997 ± 0.0002	0.996
<7>—non-linear	GU, kg	BCS and DOP	32	26.7 ± 16.4	6.00	58.2	4.92	18.4	4.14	0.952 ± 0.0073	0.906
<8>—non-linear	GU, kg	DOP	32	26.7 ± 16.4	6.00	58.2	5.36	20.0	4.41	0.944 ± 0.0077	0.891
<12>—linear	UT_np_, kg	SBW and GU	17	0.573 ± 0.15	0.325	0.884	0.1057	14.0	0.0845	0.697 ± 0.083	0.490
<13>—non-linear	UD_np_, kg	SBW and BCS	17	2.96 ± 0.91	1.66	4.17	0.356	12.0	0.263	0.896 ± 0.056	0.806
<15>—linear	EBW, kg	SBW	49	449 ± 76	269	596	9.58	2.13	7.83	0.992 ± 0.0016	0.984
<16>—non-linear	EBW, kg	SBW	49	449 ± 76	269	596	9.52	2.12	7.76	0.992 ± 0.0016	0.984

^1^SBW = shrunk body weight, GU = gravid uterus, UT_np_ = uterus of the cow in non-pregnant condition, UD_np_ = udder of the cow in non-pregnant condition, EBW = empty body weight.

^2^SD = standard error, RMSE = root mean square of error, MAE = mean of absolute error, and R = correlation between the estimated and observed values.

The RMSE and MAE were lower when GU weight was estimated from DOP and BCS (Equation <7>) than when estimated only from DOP (Equation <8>). Although the RMSE in proportion of the mean was between 18.4 and 20.0% for models used to estimate GU weight, the higher correlation (≥0.944) and R^2^ (≥0.891) observed suggests a safe application of these models to the population.

The adjusted models to predict UT_np_ and UD_np_ presented lower values of R and R^2^. This may occurred mainly due to the small dataset, since the dataset used in these models was composed only by the data from non-pregnant cows (n = 17, 12 non-pregnant and 5 from baseline group). It can be noted also that the UT_np_ and UD_np_ are quite variable when compared to the other ones used in this study. However, based on the lower standard deviation of the correlation and on the other cross-validation estimations given, the application of use of these models to the population seems still safe. Furthermore, this was the better approach found to predict these variables, which can be obtained only from studies designed for this purpose.

It can be noted that the model fitted to estimate the udder accretion due to pregnancy could not be validated by the cross-validation procedure. This model was generated using the segmented models procedures, which statistical procedure in the SAS software does not merge with the procedure used to generate replicates of the original dataset.

Overall, the validation procedure applied to the fitted statistical models showed that the use of the generated models by the population from which the sample used in this study came from (*B*. *indicus* cattle) seems to be safe. Multiple dataset, similar to the used in this study are difficult to obtain. Thus, the use of a strong statistical procedure of validation to test the produced results becomes fundamental.

### Practical usage of BW adjustments in pregnant cows

A summary of the equations and relationships generated to adjust the weights of pregnant cows to a non-pregnant status and also to establish the relationship between BW, EBW and SBW in pregnant or non-pregnant beef cows is presented in Table 6. The application of the equations in Table 6 can be a useful tool to separate how much of the total gain is attributable to maternal tissues and how much is from the pregnancy. Although the equations and relationships shown in Table 6 were made using *B*. *indicus* cattle it can be adapted for *B*. *taurus* cattle replacing some equations by those generated using *B*. *taurus* cattle. In this case, Equation <5> should be replaced by the factor 0.96 used in Beef Cattle NRC [6]; Equation <15> and <16> should be replaced by factor 0.851 used by Beef Cattle NRC [34] for mature cows; Equation <9> and <10> should be replaced by the model generated by Ferrell et al. [2, 36–39] to predict the weight of gravid uterus of pregnant *B*. *taurus* heifers [GU (g) = 19.32 × CBW × e^(0.02–0.0000143 × DOP) × DOP)^].

**Table 6:** Summary of equations used to adjust BW of pregnant and non-pregnant beef cows.

**Variable to be estimated**	**Predictors variables**	**Equation code**	**Relation**
*Non-pregnant cows*			
SBW_np_	BW	<6>	SBW = 0.8084 × BW^1.0303^
EBW_np_	SBW_np_	<15> or <16>	EBW_np_ = 0.9092 × SBW_np_, or
			EBW_np_ = 0.8424 × SBW_np_^1.0122^
*Pregnant cows*			
SBW_p_	BW	<6>	SBW = 0.8084 × BW^1.0303^
SBW_np_	SBW_p_ and PREG	-	SBW_np_ = SBW_p_ - PREG
PREG	If DOP ≤ 240: GU_dp_	-	If DOP ≤ 240: PREG = GU_dp_
	If DOP > 240: GU_dp_ and UD_dp_		If DOP > 240: PREG = GU_dp_ + UD_dp_
GU_dp_	GU and UT_np_	-	GU_dp_ = GU - UT_np_
GU	DOP or DOP and BCS	<9> or <10>	GU = 0.008010 × CBW × BCS^0.3225^ × e^((0.02544–0.0000286 × DOP) × DOP)^, or
			GU = 0.007521 × CBW × e^((0.03119–0.00004117 × DOP) × DOP)^
UT_np_	SBW_p_ and GU	<12>	If DOP ≤ 240: UT_np_ = 0.0012 × (SBW_p_ - GU + 0.6)
			If DOP > 240: UT_np_ = 0.0012 × (SBW_p_ - GU + 0.6–2)
UD_np_	SBW_p_ and BCS	<13>	UD_np_ = SBW_np_ × 0.00589 × BCS^0.2043^, or
			If DOP ≤ 240: UD_np_ = (SBW_p_ - GU_dp_) × 0.00589 × BCS^0.2043^
			If DOP > 240: UD_np_ = (SBW_p_ - GU_dp_ - 2) × 0.00589 × BCS^0.2043^
UD_dp_	UD_np_ and DOP	<14>	UD_dp_ = UD_np_ × e^((DOP - 238) × 0.0109)^ - UD_np_
EBW_p_	EBW_np_ and PREG	-	EBW_p_ = EBW_np_ + PREG
EBW_np_	SBW_np_	<15> or <16>	EBW_np_ = 0.9092 × SBW_np_, or
			EBW_np_ = 0.8424 × SBW_np_^1.0122^

A detailed example of practical application and calculation of the equations suggested in this study is presented in S1 Appendix. Also, a Microsoft Excel spreadsheet (S1 Spreadsheet) was constructed based on the equations presented in Table 6 and considerations made in this topic on BW adjustment for *B*. *taurus* cows.

### Problems related to BW adjustments in pregnant cows

It is not possible to make all the proposed weight adjustments without incurring some errors. One of the first problems is the fact that there are some circular references in the theory of the proposed equations. To try to avoid this problem we used several fixed parameters, estimated for the average of the sample of animals used to generate the proposed equations.

Another key point to consider is that the growth of the components related to the pregnancy and maternal tissues does not occur independently. During pregnancy changes occur in the maternal tissues to support nutrition and growth of the gravid uterus. Reduction in the size of the viscera [40] and mobilization of maternal body reserves tissues may be caused by the pregnancy in ruminants, although it is still a contradictory issue.

## Conclusions

The live weight of pregnant cows can be adjusted for non-pregnant condition by deduction of uterus and udder accretion weight due to pregnancy, which can be estimated as a function of days of pregnancy and shrunk body weight. Udder weight accretion as a function of pregnancy occurs after 238 d of pregnancy in Nellore cows.

## Supporting Information

S1 Spreadsheet The spreadsheet included as supporting information can be easily used to estimate the weights of cow and pregnant compound at different feeding status. The adaptation of the results from this study for *Bos taurus* or crossbred cattle was made using the information described in the section *“Practical usage of BW adjustments in pregnant cows”* of the paper. Crossbred results are average of *Bos indicus* and *Bos taurus* calculations.
(XLSX)

S1 Appendix Exemplification of practical application of the calculations proposed to adjust BW of mature Zebu cows. (DOCX)
