# Supplementary material for: Achieving Body Weight Adjustments for Feeding Status and Pregnant or Non-Pregnant Condition in Beef Cows
Source: PLoS One. 2015 Mar 20;10(3):e0112111. doi: 10.1371/journal.pone.0112111 (PMC4368534; doi:10.1371/journal.pone.0112111)
Supplement: S1 Appendix — (DOCX) [file pone.0112111.s002.docx]

**APPENDIX S1**

**Achieving body weight adjustments for feeding status and pregnant or non-pregnant condition in beef cows**

Mateus P. Gionbelli^1^*, Marcio S. Duarte^2^, Sebastião C. Valadares Filho^2^, Edenio Detmann^2^, Mario L. Chizzotti^2^, Felipe C. Rodrigues^2^, Diego Zanetti^2^, Tathyane R.S. Gionbelli^2^ and Marcelo G. Machado^2^

^1^Federal University of Lavras, Department of Animal Science, Lavras, MG, Brazil

^2^Federal University of Viçosa, Department of Animal Science, Viçosa, MG, Brazil

^*^Corresponding author: Mateus P. Gionbelli, PhD

Email: [mateus.gionbelli@dzo.ufla.br](mailto:mateus.gionbelli@dzo.ufla.br)

For exemplification of practical application an example can be used:

A pregnant cow with initial BW of 500 kg, BCS = 5 and with 120 of pregnancy, is subjected to a supposed treatment during a 150 d period; and at the end of this period is with 600 kg of BW, BCS = 6 and DOP = 270 d. The estimated CBW is 28 kg. The objective is to know the gain related to the maternal tissues and the gain related to pregnancy.

| *Initial* | *Final* |
| --- | --- |
|  |  |
| BW = 500 kg | BW = 600 kg |
| DOP = 120 d | DOP = 270 d |
| BCS = 5 | BCS = 6 |
| SBW_p_ = 0.8084 × 500^1.0303^ = 487.95 kg | SBW_p_ = 0.8084 × 600^1.0303^ = 588.78 kg |
| GU = 0.008010 × CBW × 5^0.3225^ × e^((0.02544 – 0.0000286 × 120) × 120)^ = 5.29 kg | GU = 0.008010 × CBW × 6^0.3225^ × e^((0.02544 – 0.0000286 × 270) × 270)^ = 47.80 kg |
| UT_np_ = 0.0012 × (487.95 – 5.29 + 0.6) = 0.58 kg | UT_np_ = 0.0012 × (588.78 – 47.80 + 0.6 – 2) = 0.65 kg |
| GU_dp_ = 5.29 – 0.58 = 4.71 kg | GU_dp_ = 47.80 – 0.65 = 47.15 kg |
| UD_np_ = (487.95 – 5.29) × 0.00589 × 5^0.2043^ = 3.95 kg | UD_np_ = (588.78 – 5.29 – 2) × 0.00589 × 6^0.2043^ = 4.58 kg |
| UD_dp_ = 0 kg | UD_dp_ = 4.58 × e^((270 – 238) × 0.0109 )^ – 4.58 = 1.90 kg |
| PREG = GU_dp_ = 4.71 kg | PREG = GU_dp_ + UD_dp_ = 47.15 + 1.90 = 49.05 kg |
| SBW_np_ = 487.95 – 4.71 = 483.24 kg | SBW_np_ = 588.78 – 49.05 = 539.74 kg |
| EBW_np_ = 0.8424 × 483.24^1.0122^ = 438.97 kg | EBW_np_ = 0.8424 × 539.74^1.0122^ = 490.94 kg |
| EBW_p_ = 438.97 + 4.71 = 443.67 kg | EBW_p_ = 490.94 + 49.05 = 539.99 kg |
|  |  |
| Total BW gain = 600 – 500 = 100 kg | |
| Total SBW gain = 588.78 – 487.95 = 100.83 kg | |
| Total EBW gain = 539.99 – 443.67 = 96.32 kg | |
|  | |
| ADG = 100 / 150 = 0.667 kg/d | |
| SBG = 100.83 / 150 = 0.672 kg/d | |
| EBG = 96.32 / 150 = 0.642 kg/d | |
|  | |
| Partial portions of the gain | |
|  | |
| Total PREG gain = 49.05 – 4.71 = 44.34 kg (46% of total EBG) | |
| Total maternal tissues EBW gain = 490.94 – 438.97 = 51.98 kg (54% of total EBG) | |
|  | |
| rSBG = (539.74 – 483.24) / 150 = 0.377 kg/d | |
| rEBG = (490.94 – 443.67) / 150 = 0.347 kg/d | |

where DOP = days of pregnancy, BCS = body condition score, SBW_p_ = shrunk body weight of a cow in pregnant condition, GU = gravid uterus, CBW = calf birth weight, UT_np_ = uterus plus ovaries of a cow in non-pregnant condition, GU_dp_ = accretion of gravid uterus due to pregnancy, UD_np_ = udder of a cow in non-pregnant condition, UD_dp_ = accretion of udder due to pregnancy, PREG = pregnant compound, SBW_np_ = shrunk body weight of a cow in non-pregnant condition, EBW_np_ = empty body weight of a cow in non-pregnant condition, EBW_p_ = empty body weight of a cow in pregnant condition, ADG = average daily gain, SBG = shrunk body gain, EBG = empty body gain, rSBG = real shrunk body gain (refers only to the gain of maternal tissues) and rEBG = real empty body gain (refers only to the gain of maternal tissues).
